# Co-Swarming and Local Collapse: Quorum Sensing Conveys Resilience to Bacterial Communities by Localizing Cheater Mutants in *Pseudomonas aeruginosa*

**DOI:** 10.1371/journal.pone.0009998

**Published:** 2010-04-01

**Authors:** Vittorio Venturi, Iris Bertani, Ádám Kerényi, Sergiu Netotea, Sándor Pongor

**Affiliations:** 1 International Centre for Genetic Engineering and Biotechnology, Trieste, Italy; 2 Biological Research Center of the Hungarian Academy of Sciences, Szeged, Hungary; Baylor College of Medicine, United States of America

## Abstract

**Background:**

Members of swarming bacterial consortia compete for nutrients but also use a co-operation mechanism called quorum sensing (QS) that relies on chemical signals as well as other secreted products (“public goods”) necessary for swarming. Deleting various genes of this machinery leads to cheater mutants impaired in various aspects of swarming cooperation.

**Methodology/Principal Findings:**

Pairwise consortia made of *Pseudomonas aeruginosa,* its QS mutants as well as *B. cepacia* cells show that a interspecies consortium can “combine the skills” of its participants so that the strains can cross together barriers that they could not cross alone. In contrast, deleterious mutants are excluded from consortia either by competition or by local population collapse. According to modeling, both scenarios are the consequence of the QS signalling mechanism itself.

**Conclusion/Significance:**

The results indirectly explain why it is an advantage for bacteria to maintain QS systems that can cross-talk among different species, and conversely, why certain QS mutants which can be abundant in isolated niches, cannot spread and hence remain localized.

## Introduction

It is now believed that many species of bacteria coordinate their group behavior through monitoring their population density via the production and detection of small signaling compounds in a process called quorum sensing (QS) [1], [2], [3], [4]. In crowded bacterial communities, the concentration of the secreted signals can dramatically increase resulting in a coordinated and synchronized community behavior that includes increased motility as well as the production of various “public goods” such as enzymes, surfactants, siderophores, etc (4,5). An extensive amount of work has been done in the last fifteen years highlighting that many important community phenotypes are regulated by quorum sensing. One such phenotype is the swarming movement which is a mechanism that bacterial communities use to colonize surfaces, to infect host organisms and to invade new habitats for reviews see [5], [6]. On the one hand, swarming bacteria cooperate by sharing signals and public goods, but on the other, they also compete with each other for nutrients. In other words, swarming must rely on an apparent equilibrium between co-operation and competition.

Co-operation in nature is known to be jeopardized by cheaters, and the rapid emergence of cheaters in QS communities has been recently reported in laboratory growth [7], [8] as well as *in vivo* [9]. Why do cheaters have limited success, despite their initial advantage? Are there conditions in a microbial consortium for a stable cooperation between cheaters and cooperators? These questions are closely related to the accepted notion that multi-species consortia seem to be the predominant form of life for many bacteria in nature. Members of multi-species consortia co-operate with each other via sharing signaling molecules and secreted factors, and given the frequent occurrence of multispecies consortia, the ability of different species to coexist and to tolerate each other's signals seems to be an evolutionarily stable property. But what is then the relationship between evolutionary stability of QS cooperation genes and kinetic stability of co-operating consortia? Can we engineer bacterial communities in such a way that cooperation becomes impossible?

Recently we developed a simplified computational model for describing the movement of QS bacteria on a surface [10]. Preliminary studies with this system indicated that two different bacterial models are able to swarm together under specific conditions. In order to systematically study this phenomenon *in vivo*, we assembled synthetic swarming communities from the Gram-negative bacterium *Pseudomonas aeruginosa*, its QS deficient knockout mutants, as well as its natural niche-partner species, *Burkholderia cepacia.* We found that a binary consortium can combine the skills of its participants inasmuch as it allows them to cross barriers that neither strain/species could cross alone. In contrast, deleterious mutants are excluded from consortia either by competition or by a phenomenon which we term ‘local population collapse’. According to computer simulations, both scenarios are the consequence of the QS signalling mechanism itself.

## Results

### Experimental system

**Figure 1:**
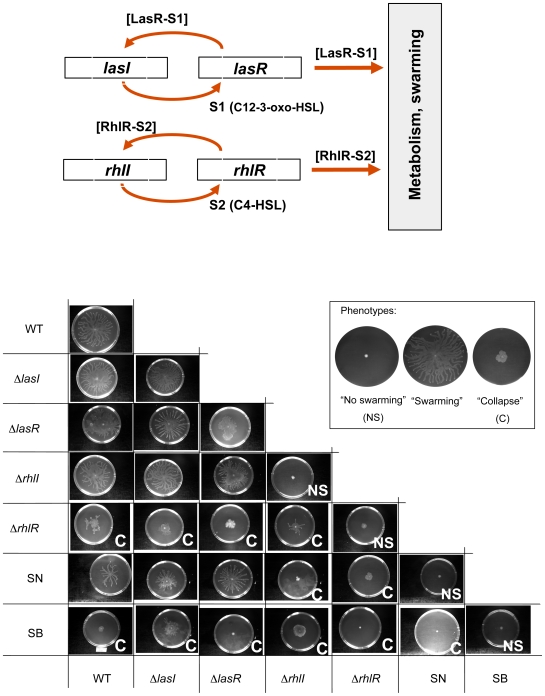
Co-swarming of *P. aeruginosa* PUPa3 and its quorum sensing mutant derivatives. **Top:** The two *N*-acyl homoserine lactone quorum sensing systems, LasI/R and RhlI/R. The *lasI* gene is responsible for the synthesis of *N*-(3-oxo-dodecanoyl)-homoserine lactone (3-oxo-C12-HSL; Figure S1) which binds and activates the cognate response regulator LasR resulting in the regulation of target gene expression. In the RhlI/R system *rhlI* directs the synthesis of *N*-(butanoyl)-homoserine lactone (C4-HSL; S2) which then interacts with the cognate RhlR influencing transcription of target genes, including those producing surfactant necessary for swarming. In *P. aeruginosa* strain PUPa3 used here, the two QS systems are not hierarchically organized [12]. **Bottom**: Swarming and co-swarming of *P. aeruginosa* PUPa3 with quorum sensing mutant derivatives. Summary of swarming movements of all possible single QS mutants and pairwise combinations of *P. aeruginosa* PUPa3. The letter C in some of the pairwise combinations refers to ‘collapse’, i.e. the consortium is not able to swarm. For experimental procedures see Figure S1. Swarming colony morphology of pure communities is shown in the diagonal.

*P. aeruginosa* is an ubiquitous Gram negative bacterium in which transcriptional regulation of many virulence and colonization-related genes is controlled by two *N*-acyl homoserine lactone (AHL)-dependent QS systems called LasI/R and RhlI/R [11](Figure 1A). In the LasI/R system, *lasI* directs the synthesis of the *N*-(3-oxo-dodecanoyl)-homoserine lactone (3-oxo-C12-HSL, S1) signal molecule which binds and activates the cognate regulator LasR resulting in the regulation of target gene expression. In the RhlI/R system on the other hand, RhlI directs the synthesis of *N*-(butanoyl)-homoserine lactone (C4-HSL, S2) which then interacts with the cognate RhlR regulator influencing transcription of target genes, including those producing surfactants such as rhamnopids that are necessary for swarming. In wild type *P. aeruginosa* (WT), both of these systems are functional and the cells form fast-growing, swarming communities that have typical branched colony morphology. The *rhl* mutants cannot swarm at all whereas the *las* mutants do swarm however less efficiently than the wild-type (see below). Deletion of any of the four genes therefore results in QS-deficient mutants that behave as obligate cheaters that are unable to fully co-operate, the resulting colony morphologies are shown in the diagonal of Figure 1B. The QS mutants could all be complemented for their swarming deficieny; the AHL synthase mutants were chemically complemented by providing the signal molecules in the media whereas the *lasR* and *rhlR* mutants were complemented with the cosmids pIB101 and pIB103 [26] respectively (data not shown). For simplicity, we classified the colony morphologies in three categories, “No Swarming”, “Swarming” and “Collapse”. The latter is an intermediate colony morphotype that results from an apparent halt of colony growth which follows after an initial growth period (explained below). Mutants deficient in the I (AHL synthase) genes do not produce the signal molecules but can respond to external signals by producing secreted factors. Mutants deficient in the R genes do not produce the secreted factors which provides them a metabolic advantage over factor-producers. So while mutants deficient in the synthase genes are information cheaters (that do not pass on the signal, but use it), mutants deficient in the R genes are public goods cheaters that do not contribute with factors necessary for swarming and growth.

It is apparent that in our *P. aeruginosa* strain, the LasI/R system has as relatively minor role in swarming since its deletion mutants can swarm on the agar plates albeit not as well as the wild-type. Deletion of the RhlI/R genes on the other hand, has a more dramatic effect on colony growth, but only the double knockout mutants (i.e. deleting both the *las* and *rhl* systems) completely loose their ability to swarm [12].

### Deletion of QS genes affects the co-swarming ability of *P. aeruginosa*

**Figure 2 pone-0009998-g002:**
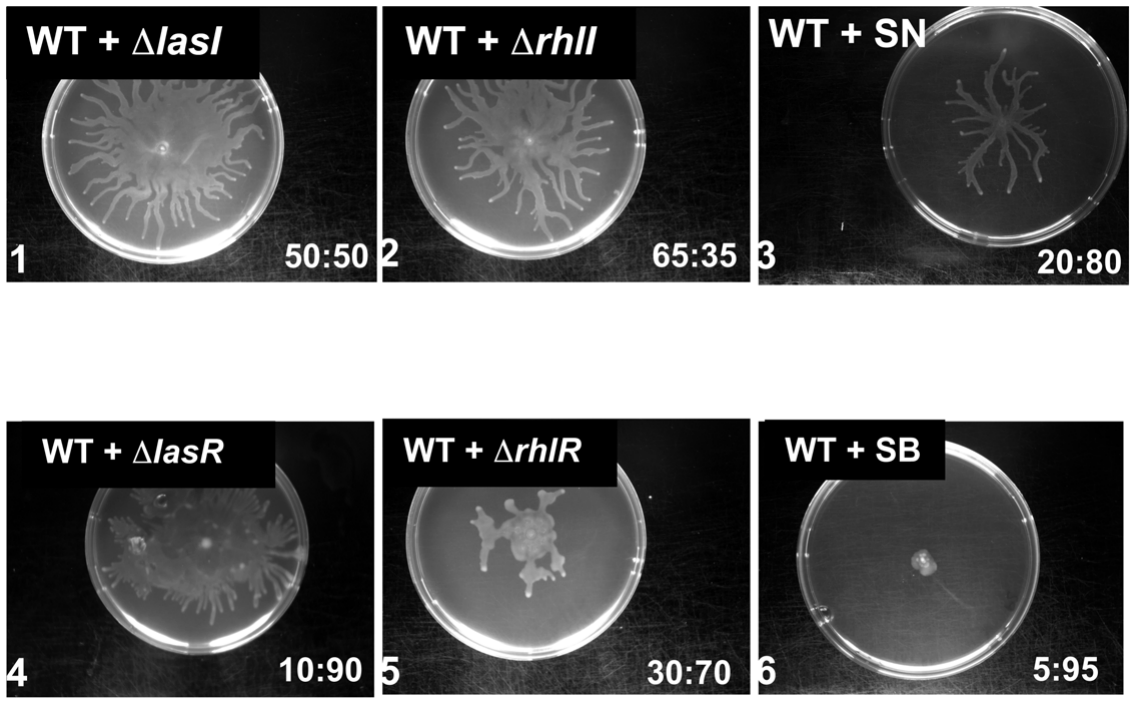
Co-swarming of *P. aeruginosa* PUPa3 with quorum sensing mutant derivatives. WT *P. aeruginosa* PUPa3 was inoculated in swarming plates in a 50∶50 ratio with the mutant strain. The final WT to mutant strain ratio is indicated in the bottom right corner of each picture, this was measured in terms of CFU counts in front-edge after 24 hours (when the photos were taken) Co-swarming of WT with single *lasI* (1), *rhlI* (2) or double *lasIrhlI* (SN; 3) mutants showed typical dendritic patterns. Co-swarming of WT with *rhlR* (5) resulted in very poor swarming movement and WT with double *rhlRlasR* (SB; 6) mutant displayed even poorer swarming resulting in a basically a non-moving bacterial consortium. Co-swarming of WT with the *lasR* mutants(4) resulted in a typical swarming pattern however the growth was slower that that of WT alone. The numbers at the bottom right of each section figure refer to a ratio of WT:mutant CFU countss measured at the front/edge of the swarming community.

*Swarming cooperation* between two strains can be studied with synthetic communities, placing 1∶1 mixtures of the strains onto swarming agar plates (Figure 1, Figure 2). The different strains in mixed swarming communities could be detected since they harbored different antibiotic markers in their genomes. It is apparent that the binary communities swarm in a way that is different, sometimes strikingly different from the single strains. For instance, mutants with a double knockout of the synthase genes (SN*, signal negative; the terms “signal negative” (SN) and “signal blind” (SB) were originally introduced by Diggle and associates [7] to designate Δ*lasI* and Δ*lasR* mutants, respectively, while we use the term for double *lasI/rhlI* and *lasR/rhlR* knock out mutants, respectively.) cannot swarm alone, but can co-swarm with the wild type albeit at a slower pace. On the other hand, double knockouts of the transcriptional regulator R genes (SB*, signal blind;), abolishes the co-swarming ability of the WT, the population collapses and growth stops. Δ*rhlI* mutants cannot swarm alone, but co-swarm well with WT, Δ*lasI* or Δ*lasR* cells. This can be explained by the fact that in these binary communities one of the partners has an intact RhlI/R system that can provide the diffusible signal for Δ*rhlI* cells. On the other hand, Δ*rhlI* and Δ*rhlR* cells cannot co-swarm, because neither of the partners have an intact RhlI/R system, since Δ*rhlR* cells do not produce enough AHL signal to allow Δ*rhlI* cells to activate the RhlI/R system [27]. We note that some of the colonies (e.g. WT- Δ*rhlR*) appeared to be intermediates between WT swarming and totally collapsed morphologies and the exact morphotype had to be determined by varying the population ratios (S2).

### Colony population kinetics protects against unwanted intruders

*Population kinetics* can be followed by counting the various cell types at different times of the run (Figure 2, Figure S1). We found in all cases that both partners can be detected in the advancing front after 24 hours, however the proportion of the partners changes during the run. In most cases, the deletion mutants constitute the majority of the population in the advancing front which roughly corresponds to 30–50 generations (Figure 2). We mention that the relative excess of the mutant is apparently proportional to the estimated metabolic advantage provided by the knockouts. Namely, deletion of R genes is known to inactivate the expression of a large number of genes, consequently, deletion mutants of the R genes (Δ*lasR*, Δ*rhlR*, and SB) have a large metabolic advantage over the WT [8], so they constitute an overwhelming majority of the co-swarming populations at 24 hours (Figure 2, panels 4–6). In contrast, the R target genes of ΔI mutants can be activated by the signals produced by the coswarming WT cells, so deletion of the I genes confers a smaller metabolic advantage to *ΔI* mutants as compared to that found in ΔR mutants. This is reflected by the fact that the proportion of ΔI mutants in ΔI:WT communities is lower than that of ΔR mutants in ΔR:WT communities.

ΔI mutants (signal cheaters) and ΔR mutants (public goods cheaters) show different co-swarming phenotypes. ΔI mutants produce branched colony morphologies similar to pure WT colonies in terms of shape and speed of growth (Figure 2A, 1–3). On the other hand, ΔR mutants give circular colonies that grow much slower than the wild type (Figure 2A, 4–7). The behavior of the two mutant classes is even more different, if we start the colonies with increasing proportion of mutants (S2, Fig S2_2). As a result, ΔR +WT colonies slow down until a virtual halt, higher proportions of mutants in the initial inoculum hastens collapse, with ΔR mutants becoming the overwhelming majority at the edge of growth. We term this behavior ‘quorum collapse’ that we define as the cessation of co-operation in a situation where resources are still available. Such a collapse seems to occur if a growth-efficient obligate cheater invades a community and monopolizes a crucial resource. In our experiments, only public goods cheaters (ΔR mutants, especially the double knockout mutant SB) collapsed the co-swarming communities, but information cheaters (ΔI mutants) were capable of stable co-swarming. In other words, certain kinds of bacteria can apparently be taken along by a swarming community, others are either gradually competed out (like BC, Figure 2B), or are localized in a population collapse (like SB, Figure 2A, 5).

From the kinetics point of view, we have seen two scenarios: i) stable swarming, which means sustainable growth of a pure or mixed colony; and ii) transient co-swarming which means that either the cheat mutants, or the co-operating WT cells are loosing in the competition. The latter (loss of WT) is quorum collapse i.e. the community falls back into a halted, stagnating phase, even though the nutrients are still in place. Importantly, the two fundamental scenarios (stable co-swarming and collapse) do not substantially depend on the WT to mutant ratios. Namely, SB produces collapse even if present as a few percentage of the starting inoculum, but the collapse will happen sooner if the initial concentration of SB is higher. In a similar way, a small percentage of SN can produce stable co-swarming, even though the steady state is reached later than it would happen with a larger percentage of SN on the starting inoculum (See S2 and the description of computational model, further down).

**Figure 3:**
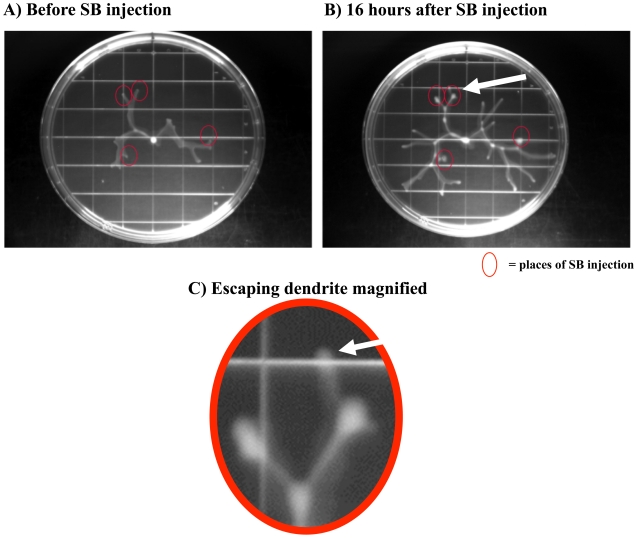
Localised collapse caused by microinjecting SB mutants into a branched colony of swarming WT *P. aeruginosa* cells. We inoculated an estimated 10^8^ CFU of SB double knockout mutant cells into moving dendrites of swarming colony of *P. aeruginosa* (A, red circles). As a result, the affected dendrites (red circles) stopped to grow while the other dendrites continued to grow normally (B). In some of the cases, the swarming community “escaped” (C, white arrow), with no or background (<1%) levels of SB cells in the escaped dendrite (The pictures in B and C were taken after 16 hours of growth after A).

Another important point to note is that our “collapsed colonies” model a localized environment. Namely, as we start the agar plate experiment, we use a well-mixed homogeneous mixture of two strains. Mixed consortia freely growing on surfaces are not well-mixed, they can be considered approximately heterogeneous only within a local – infinitesimally small – environment. So our agar-plates tend model a local environment which gradually turns into a heterogeneous environment as the colony starts to grow. The question arises whether collapse can spread within a growing, especially branched colony, or will it remain local. In order to answer this question, we microinjected SB cheater mutants into the dendrites of swarming WT *P. aeruginosa* communities (Figure 3). As a result, the affected dendrites (red circles) stopped to grow while the other, unaffected dendrites grew normally. In some of the cases, the swarming community “escaped” (white arrow), with no or background levels of SB cells in the “escaped” dendrite. So the SB mutant remains localized in both cases, either by a local collapse of the dendrite or because it is left behind by the escaping cells. Our experiments thus suggest that swarming is a local cooperation that can lead to isolation of QS cheaters. This finding indirectly explains why cheaters have limited success in moving consortia: if a collapsing mutant should arise in any of the branches, the dendrite can collapse without affecting the other branches. A similar, localized behavioral conflict has been proposed for sessile biofilm communities [13].

### Combining skills helps to overcome barriers

**Figure 4:**
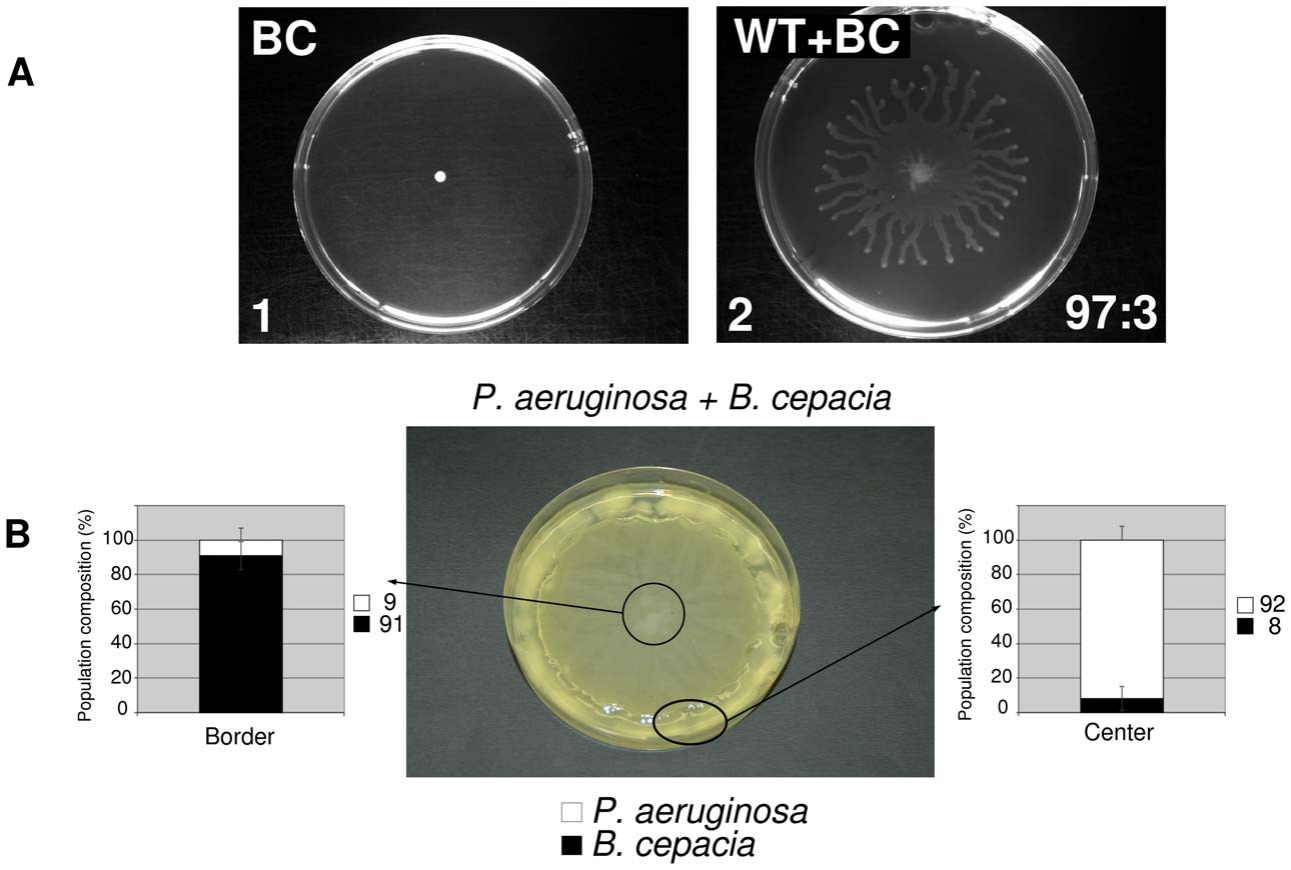
Bacteria ombining skills to help overcome barriers. **A**) *B. cepacia* cannot grow on agar plates that allow the swarming of *P. aeruginosa* (A1) Co-swarming of WT with *B. cepacia* results in a typical swarming colony morphology (A2) but growth is less efficient that of WT alone. The number at the bottom right of A2 refers to WT: *B. cepacia* ratio determined at the front-edge of the swarming community. (See S1 for all technical details of swarming assays). **B**) Co-swarming experiment between *P. aeruginosa* and *B. cepacia* in a divided plate composed of M8 agar in the central part and of rich LB medium supplemented with gentamycin 100 µg/ml in the outer rim region. In the central part of the plate, co-swarming occurs and the community is predominantly *P. aeruginosa* whereas the outer-rim is first colonized by gentamycine resistant *B. cepacia*. In a similar plate inoculated with WT *P. aeruginosa* alone results in swarming in the central part but the outer-rim in not colonized whereas when inoculated with *B. cepacia* alone, there is no swarming or colonization of the outer-rim (data not shown). *B. cepacia* is naturally resistant to gentamicine, and in this experiment, *P. aeruginosa* was labelled with the tetracycline (Tc) resistance gene which was originally introduced in order to facilitate the counting of colonies (CFUs). The counts thus indicate that the Tc and Gm resistance are present both at the beginning as well as at the end of the co-swarming phase. This fact illustrates that co-swarming in fact allows the participating species to combine their “skills” (i.e. the two antibiotic resistance phenotypes) so that the “community phenotype” of dual antibiotic resistance is preserved.

Stable co-swarming (like the one seen in WT+SN consortia) can be regarded as a way to combine the skills of the member strains. For instance, we constructed a consortium in which WT was resistant to tetracycline and SN was resistant to gentamycine, and this consortium was able to carry the gen^+^ phenotype from the centre of a swarming agar plate to the rim of the plate, so the combined gen^+^tet^+^ phenotype could be maintained. Without the quorum sensing WT, the gen+ partner SN could not have reached the rim of the plate (data not shown). Another example is a synthetic interspecies consortium formed from *P. aeruginosa* and *B. cepacia* (BC) cells. BC is a niche-mate of *P. aeruginosa* (PA) known to colonize very similar environments, such as the rhizosphere, the soil and the lung of cystic fybrosis (CF) patients [14]. BC cells cannot swarm on plates suitable for *P. aeruginosa*, however they can co-swarm with wild type PA cells (Figure 4A). The colony morphology is reminiscent of the poorer co-swarming patterns (such as WT+Δ*lasR*), however the population composition is different: in this case BC is the minor partner, and its share decreases during the run (Figure S1), however we estimate that BC is capable to co-swarm with PA for at least 30 to 50 generations In contrast we found that *E. coli* and *Chromobaceterium violaceum* cells do not migrate together with WT *P. aeruginosa*, so co-migration ability of BC cells appears to require a compatibility with PA, and this compatibility is not necessarily present in other bacterial species. This raises the possibility that even less compatible partners can co-swarm for extended periods of time which may in turn enable them to cross barriers together. The divided plate experiment shown in Figure 4B was designed to demonstrate this principle. The centre of the agar plate is swarming medium on which *B. cepacia* cannot swarm alone, while the outer rim of the plate is composed of non-swarming rich medium containing gentamycin that inhibits the growth of *P. aeruginosa* but in which *B. cepacia* can grow. In a co-swarming experiment (Figure 4B), *P. aeruginosa* will help *B. cepacia* to reach the outer compartment where *P. aeruginosa* itself cannot enter. *B. cepacia* is resistant to gentamycin which makes it possible for *P. aeruginosa* to slowly enter the outer rim of the plate. Setting up this experiment with *P. aeruginosa* alone does not allow it to enter the rim as *P. aeruginosa* is gentamcyin sensitive (data not shown).

Both examples show that consortia can combine the skills of their participant strains; both SN and BC cells depend on WT *P. aeruginosa* for swarming. This dependence allows them to join WT *P. aeruginosa* cells so a consortium will maintain a “collective phenotype” that neither of the participants possesses (eg antibiotic resistance, see above). This experiment also indicates, that even transient co-swarming can be mutually advantageous for two species by allowing them to cross barriers that neither of them is able to cross alone. This experiment also shows that a ubiquitous opportunistic pathogen can facilitate the entry of other bacteria into new habitats. For this phenomenon we use the term “combination of skills”. It is important to note that it is not the QS properties that are combined. The abilities need to be interdependent (both SN and BC depend on WT *P. aeruginosa* for moving on the QS agar plates used in these experiments) and this feature allows the consortia to combine the additional properties (e.g. antibiotic resistance) of the participating species.

### QS regulation controls population dynamics

Which known features of QS genes can be responsible for the observed behavior of QS consortia? Computer simulations can in principle provide clues how gene regulation can be linked with population dynamics data. On the other hand QS regulation, affects a substantial part of bacterial genomes [15] and analyzing the behavior of large, heterogeneous communities in such a detail would be a formidable task with too many unknowns. We thus opted for the reverse approach and asked the question: what is the simplest system that can reproduce the observed community behavior? Recently we have developed a highly simplified computational model for QS in which cell models communicate via diffusible signals that switch them from slow to fast random movement [10]. This model has a single QS system that operates as a regulatory switch regulated by the external concentrations of a single signal and a single excreted factor (S3). According to this model, colony growth can be best pictured as cells migrating within a bounded active zone in which the quantity of signals and secreted factors is sufficient to maintain swarming [10]. The driving force of this model is the balance between cooperation and competition: cells grow faster in a high density zone but must compete with more neighbors for nutrients. In this model we can produce SN or SB mutants simply by deleting the corresponding responses from the QS autoinduction regulatory circuit. [10]

**Figure 5:**
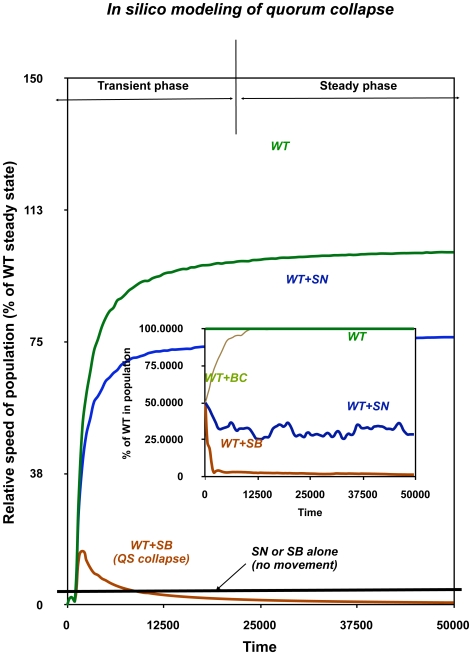
*In silico* simulation of swarming with pure and co-inoculated populations. The simulations were started with pure or 1∶1 inocula, as described in Figure S3 as well as Netotea *et al*, 2009.The speed of the front advancement and the percentage of the participating species (inset) was recorded as a function of simulation time (in arbitrary units). The swarming populations show saturation kinetics, with a rapid initial growth phase followed by a transient phase that leads to a stable steady state in which population size, composition and the speed of movement are constant. (note that on a 2D plate, steady state corresponds to steady colony growth in two dimensions).

If we use this simplified model for the analysis of mixed communities, we see essentially the same kinds of behavior that can be observed on swarming agar plates (Figure 5). Namely, 1) pure WT communities are large and grow fast. 2) WT+SB mixed communities collapse after an initial burst of growth to a very small population in which WT is a vanishing minority (Figure 3B, blue curve). 3) WT+SN communities grow at a more or less constant but relatively low speed, and have a fixed population composition. 4) Lastly, we modeled BC as a non-contributor cell type that is activated less than a WT cells so it grows marginally slower (S3). Such cells are competed out by the population by WT, and the community regains the front speed of a pure WT colony. We have also established that the computer model predicts ∼75% SN in a WT+SN consortium and ∼95% SB in a WT+SB consortium, which is in good qualitative agreement with the experimentally found values (∼73% and ∼92%, respectively, Figure S2). Obviously, the model is very simplistic, as it shows only gross differences in colony growth, but does not show differences in colony morphology. On the other hand, the model qualitatively describes the differences observed in the behaviour of the various consortia, including such non-trivial phenomena as the steady swarming of WT+SN communities vs. the collapse of WT+SB communities. The good qualitative agreement between the modeling (Figure 4) and co-swarming experiments (Figures 1–2) leave us with the conclusion that a balance between competition on the one hand, and cooperation via diffusing materials on the other, is sufficient to explain the population dynamics of pure and mixed swarming communities, and there are various forms of cooperation kinetics. Mutants that outcompete co-operators and monopolize resources can collapse the community. Inefficient strains that grow slower than cooperating cells will be outcompeted. Non-cooperating cells are thus eliminated in both cases, either by population collapse or by competition.

In summary, the computational model describes well the main experimental findings presented here (co-swarming of WT+SN, collapse of WT+SBA consortia. As deletion of any part of the QS regulatory circuit was found to abolish both co-swarming and collapse behaviour (SN+SB consortia do not swarm either *in vivo* or *in silico*), we conclude that QS regulation in itself can sufficiently explain the population dynamics of the model consortia.

## Discussion

### Implications of swarming cooperation and collapse

The experiments presented here lead to two conclusions: i) swarming collaboration allows strains to cross barriers that they could not cross alone. This phenomenon is referred to consortia “combining skills” of the participating strains inasmuch as they rescue all the phenotypes of the constituent strains, ii) secondly, cheater mutants that do not sufficiently contribute to the public goods can cause local collapse of the community so that the cheaters will be eliminated from the community. In addition, the computer modeling experiments showed that QS regulation of movement and metabolism is responsible for both phenomena so QS provides protection to consortia both against environmental challenges and against deleterious mutations. The phenomenon of QS collapse raises the possibility of using efficient QS cheats as a way to control bacterial populations which rely on QS for cooperation, growth and colonization. In fact recent experiments demonstrated that co-inoculation of WT *P. aeruginosa* and QS mutants reduced the virulence potential of *P. aeruginosa* [16]. It must be noted however that localization of cheats and the escape phenomenon observed here might render this application difficult to pursue, especially when QS is necessary for growth.

### Evolutionary benefits

We speculate that co-swarming communities, i.e. *ad hoc*, temporary coalitions of microorganisms can readily form from the thousands of bacterial species present in a natural environment. According to our simple model, mobile collaboration seems to depend only on the momentary availability of metabolic factors (signals, public goods) without necessarily considering evolutionary fixation of the genetic traits. Nevertheless, we feel that *ad hoc* formation of mobile communities may facilitate the movement of species across spatially structured habitats, so skills in inter-species collaboration may be important evolutionary traits for bacteria. There are two well-known facts that support this supposition. First, there is ample evidence for cross talk between QS species, and AHL signals are sometimes called as the “most common dialect” among Gram-negative bacteria [17]. Second, there are many proteobacteria which do not produce AHLs but possess a LuxR-family sensor/regulators [18], [19], enables them to respond to exogenous signals, in a fashion similar to WT+SN cooperation described here. The high frequency of this genotype in Proteobacteria indicates that the collaborations similar to the stable WT-SN co-operation reported here may be a frequent scenario in nature in which common molecular recognition signals enable different bacterial species to form heterogeneous consortia that cheaters cannot easily invade.

*Social dilemmas* provide intriguing possibilities for the interpretation of QS phenomena [20], [21], [22]. In our experimental set-up sustained growth of the community requires stable co-operation between cells. This situation is reminiscent of public goods games used in economy to model the behaviour of companies on the market (S4). In this game, defection pays off only on the short term, while on longer terms cooperation can emerge. This is in agreement with the present finding that public-good cheater non-cooperators (like SB) are successful only in short term (transient) scenarios. In addition, cheats collapse the community, after a transient upsurge reminiscent of economic bubbles. On the other hand, public good collaborator SN cells can stably co-swarm with a WT community, thus cooperation pays on the long term.

We note that long-term steady states observed in the simulations correspond to sustained colony growth during which the proportion of the partners and the advancement rate of the front are constant. This leaves us with the impression that sustained growth relies on equilibrium of strategies, and breaking this equilibrium may collapse the community into a stagnating state from where there is no immediate return. On the other hand, the superior swarming ability of WT colonies, observed both *in vivo* and *in silico,* confirms that co-operators are better in crossing barriers, so heterogeneities of natural environments may provide a selective advantage for quorum-sensing co-operators. This is not in contradiction with the known appearance of QS cheats in some niches, such as the presence of *P. aeruginosa* cheats in lungs of chronically infected cystic fibrosis patients [9]. Namely, opportunistic *P. aeruginosa* is so ubiquitous that its QS competence can be easily maintained in the open environment, for instance as a trait necessary for competing for hosts.

The modeling approach used in this work is based on a minimalist model. “Toy models” such as network models of metabolism, lattice models of protein folding, etc. are not expected to predict fine qualitative details rather they are used to highlight crucial mechanisms underlying a complex behavior. Our model was based on the mechanism of QS regulation controlling movement and metabolism, and the experimental phenomena studied were co-swarming and collapse of synthetic communities. These salient phenomena were correctly predicted by the model while deletion of any QS element from the model was found to abolish this behavior. The results thus suggest that QS kinetics adequately explains both collapse and co-swarming.

In summary, our experiments suggest that a balance between competition and cooperation is sufficient to explain swarming of pure and mixed bacterial communities, but there are a variety of possible outcomes even in a simple model system. Cooperation may enable heterogeneous consortia to combine the skills of different species so bacteria capable of interspecies communication may have an evolutionary advantage. On the contrary, obligate cheaters that monopolize a crucial resource can collapse the co-operation, which will immobilize the local neighborhood around them. As a result, obligate cheaters will have difficulty in overtaking a community whose survival depends on QS co-operation. The natural variability of bacteria is, in our opinion, sufficient to ensure that cheater mutants will continuously emerge and survive in isolated niches where cooperation is not as crucial as in an open environment. Finally, the results suggest that two seemingly unrelated community phenomena, resilience to environmental challenges and protection against cheaters, are the consequence of the same simple and elegant regulatory mechanism, i.e. quorum sensing [1], [2], [3], [4]. Major transitions in biology are known to produce higher level organisms via improvements in the mechanisms of information storage and transfer, as well as via the establishment of cooperative synergies alleviating competition between different units of evolution [23], [24]. QS signalling shows interesting analogies with the initial stages of this series, as QS signalling not only synchronizes the behaviour of the participating cells, but, as we have seen here, also conveys robustness to the resulting bacterial consortia.

## Materials and Methods

### *P. aeruginosa* strains, growth conditions and swarming assays

The *P. aeruginosa* PUPa3 WT strain and all QS genomic null mutants have all been described previously (Steindler et al., 2009). *P. aeruginosa* PUPa3 and derivative mutants were grown at 28°C in Luira-Bertani (LB) medium, while *B. cepacia* ATCC25416 was grown in either LB or Kings' medium (KB). Antibiotics were added when required at the following final concentrations: ampicillin 100 µg/ml, tetracycline 100 µg/ml, gentamycine 100 µg/ml and kanamycine 300 µg/ml.

Swarming assays were performed using M8 medium plates [M9 salts without NH_4_Cl; [25]] supplemented with 0.2% glucose and 0.05% glutamate and containing 0.5% agar as previously described (Murray and Kazmierczak, 2006). The inoculation was performed with a sterile toothpick dipped in a bacterial suspension of OD_600_ 2.7. Bacterial suspensions were made from single strains or combinations in different ratios. Plates were incubated at 30°C for 24 hrs.

Composition of bacterial populations, at different distances from the inoculation point on the swarming plate, were harvested from the swarming plate and diluted in LB liquid. Different dilutions of the cell suspensions were plated on LB or LB supplemented with the appropriate antibiotic. All further details are explained in the text, figure legends or Figures S1 and S2.

### Computer modeling

Computer modeling of swarming communities was carried out using randomly moving bacterial agents that produce a signal *S* and a single secreted factor *F* as described previously [10]. The details are explained in Figure S3.

## Supporting Information

Figure S1 Swarming assays and their evaluation; kinetics of population composition during co-swarming experiments. The P. aeruginosa PUPa3 QS genomic null mutants were created either via insertional mutagenesis utilizing the conjugative suicide vectors or via double homologous recombination using a marker exchange procedure. The construction of all mutants has been reported elsewhere [1]. Swarming assays were performed using M8 medium plates [M9 salts without NH4Cl; [2]] supplemented with 0.2% glucose and 0.05% glutamate and containing 0.5% agar [3]. The inoculation was performed with a sterile toothpick dipped in a bacterial suspension of OD600 2.7. Bacterial suspensions were made from a unique strain. Plates were incubated at 30°C for 24 hrs. The distribution of cells between various populations was determined by colony counting as follows. All co-swarming inoculations consisted in a 1:1 ratio of the different cell types. Plates were incubated at 30°C for 24 hrs. The sample collection and counting was determined in 5 biological replicates taking cells from the centre, middle and at the border representing fully advanced populations. Colony forming units were counted as follows: cells were harvested from the surface of the swarming plates at different distances from the inoculation point as indicated and were diluted in LB liquid medium. Different dilutions of the cell suspensions were then plated on LB or LB supplemented with the appropriate antibiotic. The results of the experiments are shown. The graphs show the composition of the population expressed as a %-age on the y-axis. References 1. Steindler L, Bertani I, De Sordi L, Schwager S, Eberl L et al. (2009) LasI/R and RhlI/R quorum sensing in an environmental strain of Pseudomonas aeruginosa. Appl Environ Microbiol 75: 5131-5140. 2. Kohler T, Curty LK, Barja F, van Delden C, Pechere JC (2000) Swarming of Pseudomonas aeruginosa is dependent on cell-to-cell signaling and requires flagella and pili. J Bacteriol 182(21): 5990-5996. 3. Murray TS, Kazmierczak BI (2006) FlhF is required for swimming and swarming in Pseudomonas aeruginosa. J Bacteriol 188(19): 6995-7004.
(0.81 MB TIF)

Figure S2 Influence of the starting population ratios. Co-swarming inoculations were performed with various ratios of the different cell types. Plates were incubated at 30°C for 24 hrs. The sample collection and counting was determined in 5 biological replicates taking cells from the centre, middle and at the border representing fully advanced populations. Colony forming units were counted as follows: cells were harvested from the surface of the swarming plates at different distances from the inoculation point as indicated and were diluted in LB liquid medium. Different dilutions of the cell suspensions were then plated on LB or LB supplemented with the appropriate antibiotic. S2_1: Note that swarming colony morphologies do not depend very strongly on the population ratio of the starting inocula, while colonies get reduced as the proportion of the non-contributor mutant is changed. S2_2: The starting and finishing mutant ratios are summarized in the table S2_3.
(2.49 MB TIF)

Figure S3 Computational model. Swarming communities were modeled with randomly moving bacterial agents that produce a signal S and an excreted factor F [1]. This is a simplified version of a colony morphology model [2] to which we added a single quorum sensing system acting as a threshold-based regulatory switch. At the beginning of the experiment, only signal S is produced, and when its external concentration exceeds a threshold, the cells increase signal production as well as start to produce factor F. When the factor concentration exceeds a threshold, the cells “switch phenotype” i.e. they speed up their movement and metabolism (S3_1). As a result, the colony starts to swarm towards locations where nutrients are available [1]. In a co-swarming simulation experiment (Figure S3_2), equal populations of bacterial cell agents (mutant and wild type) are put to the beginning of a longitudinal track that corresponds to a segment of the agar plate (A). Initially, the cells grow in place (B), then they start to swarm, and after a transient period they either reach a constant speed, or they stop swarming and remain in a stagnating state (C). The migration of the cell agents can be best pictured as mimicking the growth of a single dendrite (Figure S3_1, right panel). The position (speed) of the front, the number of “living” cell agents can be counted at every time step. The resulting values were expressed in relative terms, as a % of the corresponding values of pure WT populations. In the experiments we used the conditions for WT, SB and SN Pseudomonas aeruginosa cells described before [1]. For modeling Burkholderia cepacia, (BC) we had no parameters available, so we first used the PA models to emulate the behaviour of BC cells. The only conditions allowing this BC model to be competed off by WT PA was to decrease the growth rate of BC models (by about 10%). The three models (WT, SB, SN) did not substantially differ in this respect (Figure S3-3). Table S3_4 able summarizes the main steady state parameters of the various simulations. Sw%: percentage of swarming cells in the model population rDR: relative division rate, defined as the division rate of the mutant in the swarming state, divided by that of WT. The color code corresponds to Figure 4 of the main text. Note that higher relative division rate of the mutant is accompanied by slower population movement and smaller population size and decreased speed. rDr is a measure characterizing the relative fitness of a mutant as compared to the WT. Part S3-5 is a time course of signal and factor concentrations during co-swarming simulations. In WT+SN consortia, the level of factors is similar to that of WT consortia, but the level of signals is considerably decreased, so this consortium may be limited by the signal. Adding external signals (3-oxo-C12-HSL and C4-HSL) to the agar plates confirms this hypothesis as the level of swarming is restored to that of the wild type (data not shown). On the other hand, the collapsed WT+SB model community shows low levels of both signals and factors (Figure 5A) and this behavior cannot be restored by adding exogenous signals, either in silico, or in a laboratory experiment (data not shown). References 1. Netotea S, Bertani I, Steindler L, Venturi V, Pongor S (2008) A simple model for the early events of quorum sensing in Pseudomonas aeruginosa. Biol Direct 4: 6. 2. Ben-Jacob E, Schochet O, Tenenbaum A, Cohen I, Czirok A et al. (1994) Generic modelling of cooperative growth patterns in bacterial colonies. Nature 368(6466): 46-49.
(1.92 MB TIF)
