# Supplementary figures and images for: Co-Swarming and Local Collapse: Quorum Sensing Conveys Resilience to Bacterial Communities by Localizing Cheater Mutants in Pseudomonas aeruginosa
Source: PLoS One. 2010 Apr 1;5(4):e9998. doi: 10.1371/journal.pone.0009998 (PMC2848674; doi:10.1371/journal.pone.0009998)

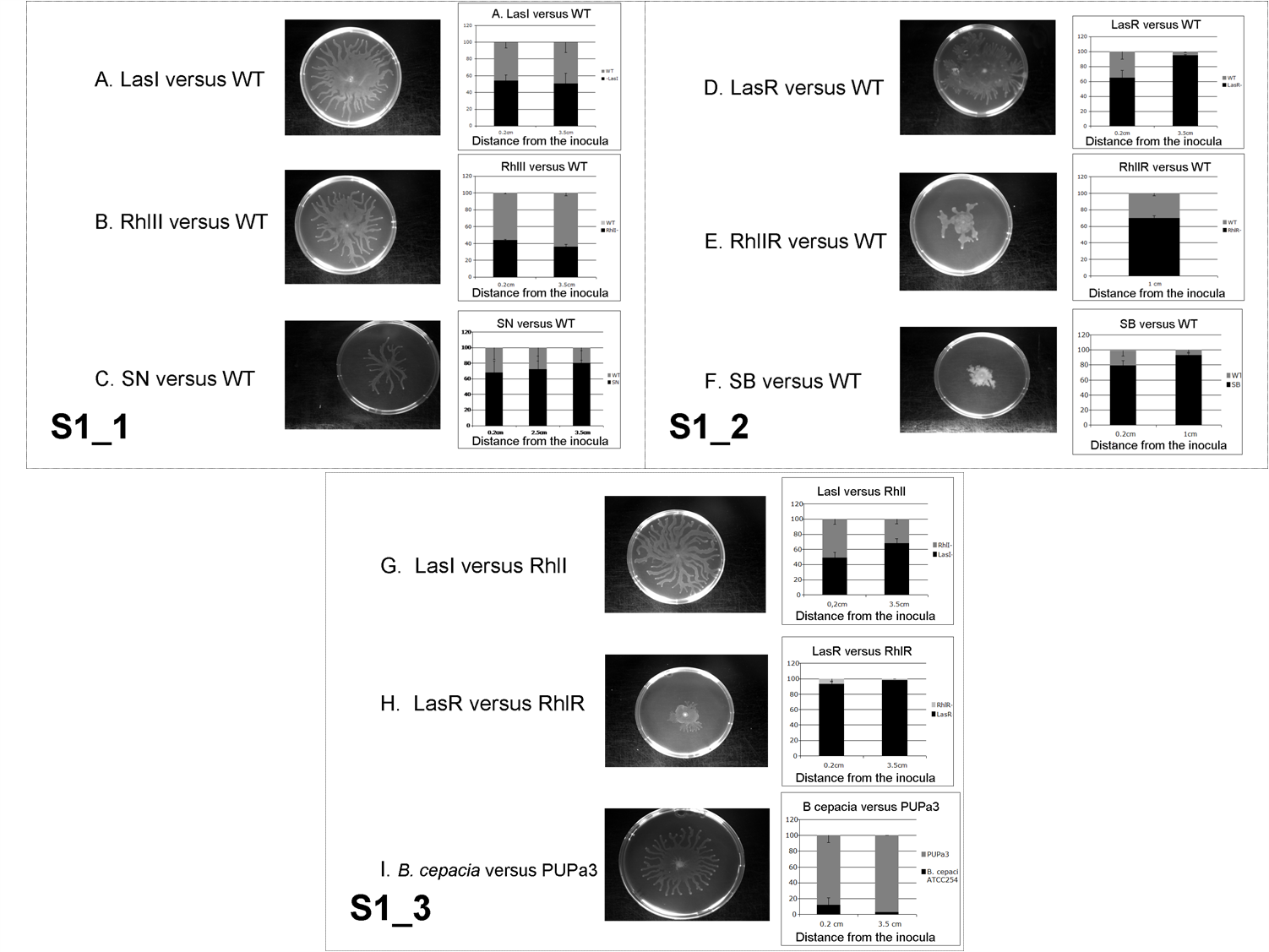

Supplement: Figure S1 — Swarming assays and their evaluation; kinetics of population composition during co-swarming experiments. The P. aeruginosa PUPa3 QS genomic null mutants were created either via insertional mutagenesis utilizing the conjugative suicide vectors or via double homologous recombination using a marker exchange procedure. The construction of all mutants has been reported elsewhere [1]. Swarming assays were performed using M8 medium plates [M9 salts without NH4Cl; [2]] supplemented with 0.2% glucose and 0.05% glutamate and containing 0.5% agar [3]. The inoculation was performed with a sterile toothpick dipped in a bacterial suspension of OD600 2.7. Bacterial suspensions were made from a unique strain. Plates were incubated at 30°C for 24 hrs. The distribution of cells between various populations was determined by colony counting as follows. All co-swarming inoculations consisted in a 1:1 ratio of the different cell types. Plates were incubated at 30°C for 24 hrs. The sample collection and counting was determined in 5 biological replicates taking cells from the centre, middle and at the border representing fully advanced populations. Colony forming units were counted as follows: cells were harvested from the surface of the swarming plates at different distances from the inoculation point as indicated and were diluted in LB liquid medium. Different dilutions of the cell suspensions were then plated on LB or LB supplemented with the appropriate antibiotic. The results of the experiments are shown. The graphs show the composition of the population expressed as a %-age on the y-axis. References 1. Steindler L, Bertani I, De Sordi L, Schwager S, Eberl L et al. (2009) LasI/R and RhlI/R quorum sensing in an environmental strain of Pseudomonas aeruginosa. Appl Environ Microbiol 75: 5131-5140. 2. Kohler T, Curty LK, Barja F, van Delden C, Pechere JC (2000) Swarming of Pseudomonas aeruginosa is dependent on cell-to-cell signaling and requires flagella and pili. J Bacteriol 182(21): 59 [file pone.0009998.s001.tif]

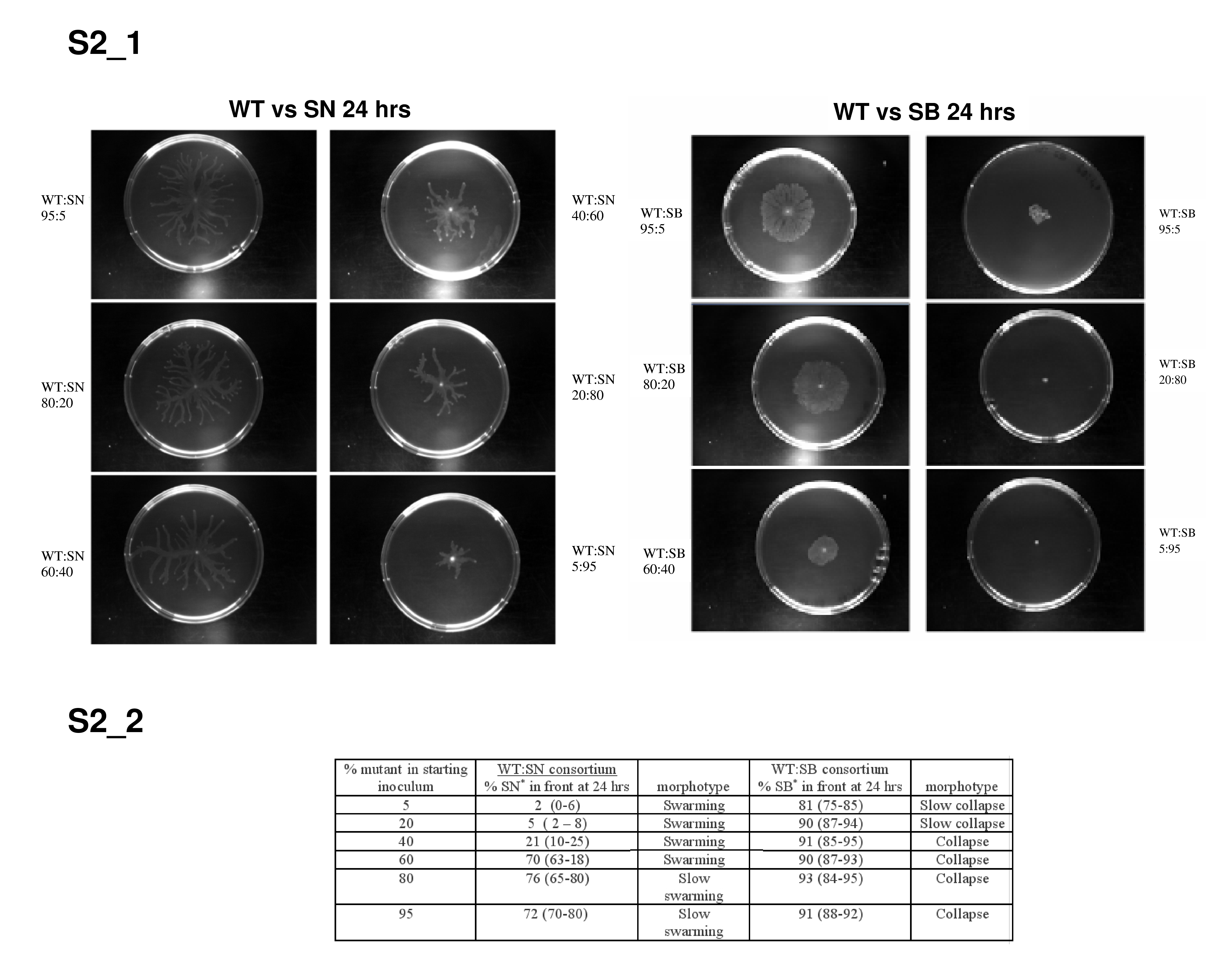

Supplement: Figure S2 — Influence of the starting population ratios. Co-swarming inoculations were performed with various ratios of the different cell types. Plates were incubated at 30°C for 24 hrs. The sample collection and counting was determined in 5 biological replicates taking cells from the centre, middle and at the border representing fully advanced populations. Colony forming units were counted as follows: cells were harvested from the surface of the swarming plates at different distances from the inoculation point as indicated and were diluted in LB liquid medium. Different dilutions of the cell suspensions were then plated on LB or LB supplemented with the appropriate antibiotic. S2_1: Note that swarming colony morphologies do not depend very strongly on the population ratio of the starting inocula, while colonies get reduced as the proportion of the non-contributor mutant is changed. S2_2: The starting and finishing mutant ratios are summarized in the table S2_3. (2.49 MB TIF) [file pone.0009998.s002.tif]

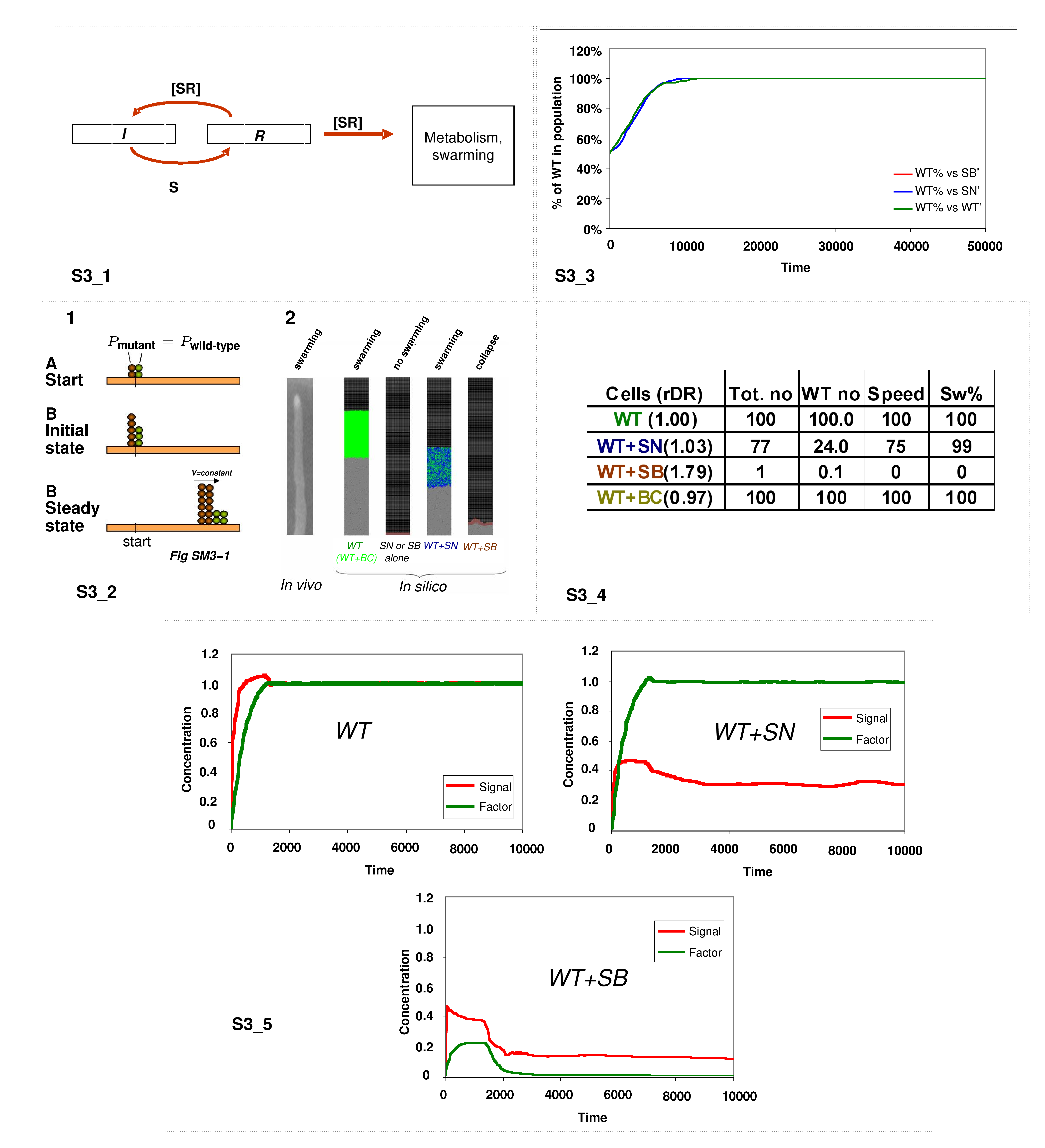

Supplement: Figure S3 — Computational model. Swarming communities were modeled with randomly moving bacterial agents that produce a signal S and an excreted factor F [1]. This is a simplified version of a colony morphology model [2] to which we added a single quorum sensing system acting as a threshold-based regulatory switch. At the beginning of the experiment, only signal S is produced, and when its external concentration exceeds a threshold, the cells increase signal production as well as start to produce factor F. When the factor concentration exceeds a threshold, the cells “switch phenotype” i.e. they speed up their movement and metabolism (S3_1). As a result, the colony starts to swarm towards locations where nutrients are available [1]. In a co-swarming simulation experiment (Figure S3_2), equal populations of bacterial cell agents (mutant and wild type) are put to the beginning of a longitudinal track that corresponds to a segment of the agar plate (A). Initially, the cells grow in place (B), then they start to swarm, and after a transient period they either reach a constant speed, or they stop swarming and remain in a stagnating state (C). The migration of the cell agents can be best pictured as mimicking the growth of a single dendrite (Figure S3_1, right panel). The position (speed) of the front, the number of “living” cell agents can be counted at every time step. The resulting values were expressed in relative terms, as a % of the corresponding values of pure WT populations. In the experiments we used the conditions for WT, SB and SN Pseudomonas aeruginosa cells described before [1]. For modeling Burkholderia cepacia, (BC) we had no parameters available, so we first used the PA models to emulate the behaviour of BC cells. The only conditions allowing this BC model to be competed off by WT PA was to decrease the growth rate of BC models (by about 10%). The three models (WT, SB, SN) did not substantially differ in this respect (Figure S3-3). Table S3_4 able summarizes the main s [file pone.0009998.s003.tif]
